# Epidemiology and antimicrobial resistance of pathogens in pediatric sinus infections: a retrospective study at a Japanese otolaryngology clinic (2023–2025)

**DOI:** 10.3389/fcimb.2025.1662544

**Published:** 2025-09-15

**Authors:** Shiori Kitaya, Toshiaki Kikuchi, Kazuhiro Nomura, Yuri Nomura, Ryoukichi Ikeda, Hajime Kanamori, Yukio Katori

**Affiliations:** ^1^ Department of Otolaryngology-Head and Neck Surgery, Tohoku University Graduate School of Medicine, Sendai, Miyagi, Japan; ^2^ Department of Infectious Diseases and Laboratory Medicine, Kanazawa University, Kanazawa, Ishikawa, Japan; ^3^ Sendai Sta. North Gate ENT. Clinic, Sendai, Miyagi, Japan; ^4^ Center for Otologic Surgery, Sen-en Rifu Hospital, Sendai, Miyagi, Japan; ^5^ Department of Otolaryngology-Head and Neck Surgery, Iwate Medical University, School of Medicine, Shiwa, Iwate, Japan

**Keywords:** pediatric rhinosinusitis, antimicrobial resistance, *Haemophilus influenzae*, *Streptococcus pneumoniae*, *Staphylococcus aureus*

## Abstract

**Introduction:**

*Haemophilus influenzae* and *Streptococcus pneumoniae* are two of the major pathogens responsible for pediatric rhinosinusitis. Rising antimicrobial resistance (AMR) and pneumococcal serotype replacement have complicated treatment decisions. This study aimed to investigate bacterial distribution and AMR patterns in nasal discharge samples from children at a Japanese otolaryngology clinic.

**Methods:**

We conducted a retrospective study at an otolaryngology clinic in Sendai, Japan, from February 2023 to March 2025. A total of 2009 nasal discharge specimens were analyzed. Bacterial identification and antimicrobial susceptibility testing were performed according to Clinical and Laboratory Standards Institute guidelines. *H. influenzae* and *S. pneumoniae* were phenotypically classified and stratified by age (0-2, 3-5, and 6-13 years). Age-group comparisons were performed using Fisher’s exact test with Holm correction.

**Results:**

Pathogens were detected in 1862 samples (92.7%). The most frequently isolated organisms were *Moraxella catarrhalis* (30.9%), *H. influenzae* (23.0%), and *S. pneumoniae* (20.6%). Among the 697 *H. influenzae* isolates, 44.8% were ampicillin-resistant, and 31.3% of all isolates were β-lactamase-negative ampicillin-resistant (BLNAR) strains. Some BLNAR strains exhibited reduced susceptibility to amoxicillin–clavulanic acid (MIC_90_ = 8 μg/mL). Cefotaxime, cefditoren, and levofloxacin remained highly active. Among the 625 *S. pneumoniae* isolates, 66.6% were penicillin-susceptible, 31.0% were intermediate, and 2.4% were resistant; resistance to clarithromycin was observed in 84.3% of isolates. The prevalence of *Staphylococcus aureus* increased with age, with 25% of isolates in the 6-13-year group identified as methicillin-resistant.

**Conclusion:**

*H. influenzae* and *S. pneumoniae* remain key pathogens in pediatric rhinosinusitis and exhibit high AMR rates. Age-specific trends, including increased methicillin-resistant *S. aureus* in older children, should guide empiric therapy. Ongoing AMR surveillance and culture-based management are essential.

## 1 Introduction

Rhinosinusitis and related otorhinolaryngologic infections are common in both children and adults, often developing as bacterial complications of viral upper respiratory tract infections. The causative pathogens of rhinosinusitis differ between pediatric and adult populations. In children, *Haemophilus influenzae*, *Streptococcus pneumoniae*, and *Moraxella catarrhalis* are most frequently implicated (Sawada and Matsubara, 2021). Over the past two decades, the emergence and spread of antimicrobial resistance among otorhinolaryngologic pathogens has become a major public health concern. In Japan, the prevalence of penicillin-resistant *S. pneumoniae* (PRSP) and ampicillin-resistant *H. influenzae*—particularly β-lactamase-negative ampicillin-resistant (BLNAR) strains—increased significantly during the 2000s (Suzuki et al., 2020). To prevent severe pneumococcal infections, Japan introduced the 7-valent pneumococcal conjugate vaccine (PCV7) in April 2013, followed by the 13-valent vaccine (PCV13) in November of the same year. Although the prevalence of vaccine-type strains declined sharply, non-vaccine, multidrug-resistant serotypes such as 23A, 15A, and 35B have since emerged as dominant (Kawaguchiya et al., 2024). To address this, newer conjugate vaccines—PCV15 (approved for children in June 2023) and PCV20 (approved in March 2024)—have been introduced and are expected to enhance serotype coverage. In contrast, no widely used vaccine exists for non-typeable *H. influenzae*, making antimicrobial stewardship critical for managing resistance. National surveillance data show that the prevalence of BLNAR strains remains high, at approximately 40-50% (Yamada et al., 2020).

Thus, otorhinolaryngologic infections, particularly rhinosinusitis, represent an especially important disease group in children. Among them, acute otitis media (AOM) is one of the most common pediatric infections and often develops as a complication of viral upper respiratory tract infections. By school age, approximately 80-90% of children experience otitis media, with *S. pneumoniae*, non-typeable *H. influenzae*, and *M. catarrhalis* being the predominant causative pathogens. The pathogenesis of AOM shares several mechanisms with rhinosinusitis, most notably contiguous mucosal inflammation and Eustachian tube dysfunction. Furthermore, because the nasopharynx and middle ear are anatomically connected via the Eustachian tube and harbor overlapping bacterial pathogens, a strong clinical and microbiological association between the two diseases has been demonstrated (Xu et al., 2019; Silva et al., 2021).

In response, the 2024 revision of the Japanese Pediatric AOM Guidelines recommended high-dose amoxicillin as the first-line therapy, and the position of watchful waiting was reiterated as a strategy to reduce the selective pressure for antimicrobial resistance (The Japanese Society for Pediatric Otorhinolaryngology, 2024). Nevertheless, despite these vaccination and guideline-based interventions, up-to-date epidemiological data from routine clinical settings remain scarce.

Therefore, we conducted this study using nasal discharge culture results collected from an otolaryngology clinic in Sendai between 2022 and 2025 to clarify the distribution of bacterial species and recent trends in antimicrobial susceptibility in the context of new vaccine implementation and evolving clinical practice guidelines.

## 2 Materials and methods

### 2.1 Study design and setting

We conducted a retrospective observational study using microbiological data from nasal discharge cultures and clinical information from patients treated at the Sendai sta. North Gate ENT. Clinic in Miyagi Prefecture, Japan. This clinic provides outpatient otolaryngologic care to patients of all ages. The study period spanned from February 2023 to March 2025. All nasal discharge specimens submitted for bacterial culture during this period were included in the analysis. These specimens were typically collected from patients presenting with symptoms of rhinosinusitis or persistent rhinorrhea suspected to be of bacterial origin. Patients who underwent fungal cultures only or viral testing only were excluded. Clinical information, including age and sex, was extracted from the patients’ medical records. When multiple specimens were collected from the same patient during the study period, each specimen was treated as an independent observational unit. Additionally, if the same bacterial species was repeatedly isolated from a single patient, each isolate was considered a separate episode if it was determined to represent a distinct infectious event, such as a recurrence. The primary outcomes of this study were the distribution of bacterial species isolated and the proportion of isolates exhibiting key antimicrobial resistance phenotypes.

### 2.2 Microbiological analysis

Nasal discharge specimens were submitted to the microbiology laboratory of BML Inc., a commercial facility located in Saitama Prefecture, Japan, where they were processed using standard bacterial culture techniques. Samples were inoculated onto appropriate media (e.g., blood agar, chocolate agar) and incubated under conditions suitable for both aerobic and facultative organisms. Bacterial identification was primarily performed using conventional methods, including colony morphology, hemolysis patterns, Gram staining, and biochemical testing. *S. pneumoniae* was identified based on α-hemolytic colonies, characteristic morphology, and optochin susceptibility. *H. influenzae* was identified by its colony appearance and its requirement for both X and V growth factors. *Staphylococcus aureus* was confirmed based on colony morphology and with automated identification systems such as the WalkAway^®^ DXM1096 system (Beckman Coulter, Brea, CA, USA) and the VITEK^®^ 2 XL (blue) system (bioMérieux, Marcy-l’Étoile, France). When conventional or automated methods were insufficient, mass spectrometry–based platforms such as the VITEK^®^ MS system (bioMérieux) or MALDI Biotyper Sirius system (Bruker Daltonics, Bremen, Germany) were used for definitive identification. Isolates considered part of the normal commensal flora (e.g., coagulase-negative staphylococci, *Corynebacterium* spp.) were excluded as causative pathogens. When more than one potential pathogen was isolated from a specimen, each isolate was included in the frequency analysis.

Antimicrobial susceptibility testing was performed using the broth microdilution method with either the WalkAway^®^ DXM1096 system (Beckman Coulter, Brea, CA, USA) or the VITEK^®^ 2 XL (blue) system (bioMérieux, Marcy-l’Étoile, France), in accordance with the Clinical and Laboratory Standards Institute (CLSI) guidelines (M100-S26 and 29th edition) (Clinical and Laboratory Standards Institute, 2019). Minimum inhibitory concentrations (MICs) and susceptibility breakpoints were determined for *H. influenzae* and *S. pneumoniae*. For *S. pneumoniae*, the antimicrobial agents tested included penicillin G, amoxicillin–clavulanic acid (2:1 ratio), cefotaxime, cefditoren, levofloxacin, clarithromycin, vancomycin, and meropenem. For *H. influenzae*, the agents tested were ampicillin, amoxicillin–clavulanic acid (2:1 ratio), cefotaxime, cefditoren, levofloxacin, clarithromycin, and meropenem. *S. pneumoniae* isolates were categorized based on CLSI susceptibility breakpoints for oral penicillin V in non-meningitis respiratory infections as follows: susceptible (MIC ≤ 0.06 μg/mL), intermediate (MIC = 0.12-1 μg/mL), and resistant (MIC ≥ 2 μg/mL). In this study, isolates interpreted as intermediate or resistant were considered penicillin-nonsusceptible *S. pneumoniae*. *H. influenzae* isolates were classified into five categories according to CLSI criteria (Clinical and Laboratory Standards Institute, 2019): β-lactamase–nonproducing ampicillin-susceptible (BLNAS; MIC of ampicillin ≤ 1 μg/mL), β-lactamase–nonproducing ampicillin-intermediately resistant (low-BLNAR; MIC of ampicillin = 2 μg/mL), β-lactamase–nonproducing ampicillin-resistant (BLNAR; MIC of ampicillin ≥ 4 μg/mL), β-lactamase–producing ampicillin-resistant (BLPAR; MIC of amoxicillin–clavulanic acid ≤4 mg/L), β-lactamase–producing amoxicillin–clavulanic acid–resistant (BLPACR; MIC of amoxicillin–clavulanic acid ≥8 mg/L). β-lactamase production was confirmed using the nitrocefin test (Showa Chemical, Tokyo, Japan). *S. aureus* isolates were defined as methicillin-resistant *S. aureus* (MRSA) if they exhibited resistance to either oxacillin or cefoxitin.

### 2.3 Statistical analysis

The results were expressed as medians with interquartile ranges or as proportions relative to the total number of patients or isolates. Differences in the distribution of bacterial species among the three age groups (0-2, 3-5, and 6-13 years) were analyzed using Fisher’s exact test for categorical variables. When significant differences were observed, pairwise comparisons between age groups were conducted using the Steel–Dwass test. *p*-values were adjusted for multiple comparisons using the Holm method. All statistical analyses were performed using GraphPad Prism version 10 (GraphPad Software, San Diego, CA, USA).

### 2.4 Ethical considerations

This study was conducted in accordance with the principles of the Declaration of Helsinki and the Ethical Guidelines for Medical and Biological Research Involving Human Subjects issued by the Japanese government. The study protocol was reviewed and approved by the Institutional Review Board of Sen-en Rifu Hospital (Approval No. 20250408). Given the retrospective nature of the study and the use of de-identified culture data, the requirement for informed consent was waived by the Institutional Review Board.

## 3 Results

### 3.1 Patient and sample characteristics

Between February 2023 and March 2025, a total of 2009 nasal discharge specimens were collected. The demographic and clinical characteristics of patients who underwent nasal discharge culture are summarized in **Table 1**. The median age was 4 years (interquartile range: 2-6 years). Most patients were young children, with 32.6% aged 0-2 years, 35.2% aged 3-5 years, and 32.2% aged 6-13 years. There was a slight male predominance: 1140 males (56.7%) and 869 females (43.3%).

**Table 1:** Demographic and clinical features of patients sampled for nasal discharge culture.

Variable (n = 2009)	Total, n (%)
Age (years)	
Median (Interquartile range)	4 (2-6)
Age category (years)	
0–2	655 (32.6)
3–5	707 (35.2)
6–13	647 (32.2)
Gender	
Male	1140 (56.7)
Female	869 (43.3)
Nasal discharge culture results	
Positive	1862 (92.7)
Negative	147 (7.3)

Data are presented as numbers (%) unless indicated otherwise.

Of the 2009 specimens, 1862 (92.7%) yielded one or more bacterial species with pathogenic potential, while 147 (7.3%) were either culture-negative or grew only normal nasal flora. In total, 3034 bacterial isolates were identified. The most frequently identified organism was *M. catarrhalis* (936 isolates, 30.9%), followed by *H. influenzae* (697 isolates, 23.0%) and *S. pneumoniae* (625 isolates, 20.6%). *S. aureus* accounted for 473 isolates (15.6%), other *Moraxella* species for 210 isolates (6.9%), and *Streptococcus pyogenes* for 64 isolates (2.1%). *Pseudomonas aeruginosa* was detected in 5 isolates (0.2%).

### 3.2 Antimicrobial susceptibility patterns of *H. influenzae* and *S. pneumoniae*

The antimicrobial susceptibility profiles of *H. influenzae* (n = 697) and *S. pneumoniae* (n = 625) are summarized in **Table 2**. Among *H. influenzae* isolates, 44.8% were classified as ampicillin-resistant, comprising 31.3% BLNAR, 12.5% BLPAR, and 1.0% BLPACR strains. Among BLNAR isolates, 29.4% were resistant to amoxicillin–clavulanic acid (MIC_90_ = 8 μg/mL). Cefotaxime and cefditoren remained highly effective, with over 90% of isolates susceptible regardless of resistance phenotype. Levofloxacin demonstrated activity against virtually all isolates, while clarithromycin non-susceptibility was more variable, observed in 17.4% of BLNAR, 21.8% of BLPAR, and 28.6% of BLPACR strains. Meropenem showed relatively potent activity against all isolates (MIC_90_ ≤ 0.5 μg/mL). Among *S. pneumoniae* isolates, penicillin-susceptible strains (PSSP) accounted for 66.6%, penicillin-intermediate strains (PISP) for 31.0%, and PRSP for 2.4%. Almost all PSSP and PISP strains were fully susceptible to amoxicillin-clavulanate, cefotaxime, and cefditoren. However, among PRSP isolates, reduced susceptibility to cefditoren was observed (MIC_90_ = 1 μg/mL; 33.3% intermediate). Notably, 84.3% of all *S. pneumoniae* isolates were resistant to clarithromycin, with resistance particularly high among PISP (97.0%) and PRSP (100%) strains. In contrast, levofloxacin and vancomycin retained high activity, with susceptibility rates exceeding 95%.

**Table 2:** Antimicrobial susceptibility profiles of *Haemophilus influenzae* and *Streptococcus pneumoniae*.

Organism/Antimicrobial agent	Resistance class	n (isolates)	Susceptible	Intermediate	Resistant	MIC_50_	MIC_90_	MIC range
*Haemophilus influenzae*								
Ampicillin	BLNAS	238	238 (100)	0	0	0.25	1	≤0.12-1
	low–BLNAR	147	0	147 (100)	0	2	2	2
	BLNAR	218	0	0	218 (100)	4	≥8	4-≥8
	BLPAR	87	0	0	87 (100)	≥8	≥8	≥8
	BLPACR	7	0	0	7 (100)	≥8	≥8	≥8
	Total	697	238 (34.1)	147 (21.1)	312 (44.8)	2	≥8	≤0.12-8
Amoxicillin–clavulanic acid	BLNAS	238	238 (100)	0	0	≤1	≤1	≤1-4
	low–BLNAR	147	145 (98.6)	0	2 (1.4)	2	4	≤1-8
	BLNAR	218	154 (70.6)	0	64 (29.4)	4	8	≤1-≥16
	BLPAR	87	87 (100)	0	0	≤1	4	≤1-4
	BLPACR	7	0	0	7 (100)	8	8	8
	Total	697	456 (65.4)	0	241 (34.6)	2	8	≤1-≥16
*Haemophilus influenzae*								
Cefotaxime	BLNAS	238	238 (100)	0	0	≤0.25	≤0.25	≤0.25-1
	low–BLNAR	147	143 (97.3)	4 (2.7)	0	0.5	1	≤0.25-2
	BLNAR	218	199 (91.3)	0	19 (8.7)	1	1	≤0.25-2
	BLPAR	87	87 (100)	0	0	≤0.25	0.5	≤0.25-1
	BLPACR	7	7 (100)	0	0	1	1	0.5-1
	Total	697	674 (96.7)	23 (3.3)	0	0.5	1	≤0.25-2
Cefditoren	BLNAS	238	238 (100)	0	0	≤0.03	≤0.03	≤0.03-0.25
	low–BLNAR	147	147 (100)	0	0	0.12	0.25	≤0.03-0.5
	BLNAR	218	218 (100)	0	0	0.25	0.25	≤0.03-0.5
	BLPAR	87	87 (100)	0	0	≤0.03	0.12	≤0.03-0.5
	BLPACR	7	7 (100)	0	0	0.25	0.25	0.12-0.25
	Total	697	697 (100)	0	0	0.12	0.25	≤0.03-0.5
Levofloxacin	BLNAS	238	238 (100)	0	0	≤0.25	≤0.25	≤0.25-1
	low–BLNAR	147	147 (100)	0	0	≤0.25	≤0.25	≤0.25-1
	BLNAR	218	216 (99.1)	2 (0.9)	0	≤0.25	≤0.25	≤0.25-2
	BLPAR	87	87 (100)	0	0	≤0.25	≤0.25	≤0.25-0.5
	BLPACR	7	7 (100)	0	0	0.25	1	0.25-1
	Total	697	695 (99.7)	2 (0.3)	0	≤0.25	≤0.25	≤0.25-2
Clarithromycin	BLNAS	238	207 (87.0)	29 (12.2)	2 (0.8)	8	16	≤4-≥64
	low–BLNAR	147	128 (87.1)	18 (12.2)	1 (0.7)	8	16	≤4-32
	BLNAR	218	180 (82.6)	38 (17.4)	0	8	16	≤4-16
	BLPAR	87	68 (78.2)	15 (17.2)	4 (4.6)	8	16	≤4-≥64
	BLPACR	7	5 (71.4)	0	2 (28.6)	8	≥64	8-≥64
	Total	697	587 (84.2)	101 (14.5)	9 (1.3)	8	16	≤4-≥64
Meropenem	BLNAS	238	238 (100)	0	0	≤0.06	0.12	≤0.06-0.25
	low–BLNAR	147	145 (98.6)	2 (1.4)	0	0.12	0.25	≤0.06-0.5
	BLNAR	218	178 (81.7)	40 (18.3)	0	0.25	0.5	≤0.06-0.5
	BLPAR	87	87 (100)	0	0	≤0.06	0.25	≤0.06-0.25
	BLPACR	7	5 (71.4)	2 (28.6)	0	0.25	0.5	0.25-0.5
	Total	697	653 (93.7)	44 (6.3)	0	≤0.06	0.25	≤0.06-0.5
*Streptococcus pneumoniae*								
Penicillin G	PSSP	416	416 (100)	0	0	≤0.03	0.06	≤0.03-0.06
	PISP	194	0	194 (100)	0	0.5	1	0.12-1
	PRSP	15	0	0	15 (100)	2	4	2-4
	Total	625	416 (66.6)	194 (31.0)	15 (2.4)	0.06	1	≤0.03-4
Amoxicillin–clavulanic acid	PSSP	416	416 (100)	0	0	≤0.25	≤0.25	≤0.25
	PISP	194	194 (100)	0	0	1	1	≤0.25-2
	PRSP	15	9 (60.0)	6 (40.0)	0	2	4	1-4
	Total	625	619 (99.0)	6 (1.0)	0	≤0.25	1	≤0.25-4
Cefotaxime	PSSP	416	416 (100)	0	0	0.12	0.5	≤0.06-0.5
	PISP	194	193 (99.5)	1 (0.5)	0	0.25	0.5	≤0.06-2
	PRSP	15	15 (100)	0	0	1	1	≤0.25-1
	Total	625	624 (99.8)	1 (0.2)	0	0.25	0.5	≤0.06-2
Cefditoren	PSSP	416	416 (100)	0	0	≤0.12	≤0.12	≤0.12-0.25
	PISP	194	194 (100)	0	0	≤0.12	0.25	≤0.12-0.5
	PRSP	15	10 (66.7)	5 (33.3)	0	0.5	1	0.25-1
	Total	625	620 (99.2)	5 (0.8)	0	≤0.12	0.25	≤0.12-1
Levofloxacin	PSSP	416	414 (99.5)	0	2 (0.5)	1	1	≤0.5-≥8
	PISP	194	185 (95.4)	0	9 (4.6)	1	1	≤0.5-≥8
	PRSP	15	15 (100)	0	0	1	1	≤0.5-1
	Total	625	614 (98.2)	0	11 (1.8)	1	1	≤0.5-≥8
Clarithromycin	PSSP	416	86 (20.7)	6 (1.4)	324 (77.9)	≥2	≥2	≤0.12-≥2
	PISP	194	3 (1.5)	3 (1.5)	188 (97.0)	≥2	≥2	≤0.12-≥2
	PRSP	15	0	0	15 (100)	≥2	≥2	≥2
	Total	625	89 (14.2)	9 (1.5)	527 (84.3)	≥2	≥2	≤0.12-≥2
Vancomycin	PSSP	416	416 (100)	0	0	≤0.25	0.5	≤0.25-1
	PISP	194	194 (100)	0	0	≤0.25	0.5	≤0.25-0.5
	PRSP	15	15 (100)	0	0	≤0.25	0.5	≤0.25-1
	Total	625	625 (100)	0	0	≤0.25	0.5	≤0.25-1
Meropenem	PSSP	416	416 (100)	0	0	≤0.12	≤0.12	≤0.12
	PISP	194	156 (80.4)	38 (19.6)	0	0.25	0.5	≤0.12-0.5
	PRSP	15	2 (13.3)	13 (86.7)	0	0.5	0.5	0.25-0.5
	Total	625	574 (91.8)	51 (8.2)	0	≤0.12	0.25	≤0.12-0.5

MIC_50_, MIC that inhibited growth of 50% of the isolates. MIC_90_, MIC that inhibited growth of 90% of the isolates. MIC values are in mg/L.

BLNAR, β-lactamase-negative ampicillin-resistant; BLNAS, β-lactamase-negative ampicillin-susceptible; BLPAR, β-lactamase-producing ampicillin-resistant; BLPACR, β-lactamase–producing amoxicillin–clavulanic acid–resistant; MIC, minimal inhibitory concentration; PISP, penicillin-intermediate *Streptococcus pneumoniae*; PRSP, penicillin-resistant *S. pneumoniae*; PSSP, penicillin-susceptible *S. pneumoniae*.

### 3.3 Age-specific distribution of bacterial isolates and antimicrobial susceptibility of *H. influenzae* and *S. pneumoniae*

The antimicrobial susceptibility profiles of *H. influenzae* and *S. pneumoniae* varied significantly by age group (**Table 3**). Among the 625 *S. pneumoniae* isolates, PSSP accounted for 54.8% in the 0-2-year age group, 69.8% in the 3-5-year group, and 80.3% in the 6-13-year group, showing a significant increasing trend with age (*p* < 0.0001). After multiple comparisons correction, significant differences were observed between the 3-5 and 6-13-year groups (*p* = 0.0002), and between the 0-2 and 6-13-year groups (*p* < 0.0001). Conversely, PISP were more frequently detected in younger children, accounting for 41.5% in the 0-2-year group, 28.5% in the 3-5-year group, and 18.4% in the 6-13-year group (*p* < 0.0001). Adjusted *p*-values confirmed significant differences between the 3-5 and 6-13-year groups (*p* = 0.013), as well as between the 0-2 and 6-13-year groups (*p* = 0.0007). PRSP were infrequently detected across all age groups. In contrast, no significant age-related differences were observed in the antimicrobial susceptibility categories of *H. influenzae*.

**Table 3:** Age distribution of *Haemophilus influenzae* and *Streptococcus pneumoniae* Isolates by antimicrobial susceptibility classification.

Bacterial species	0–2 y	3–5 y	6–13 y	All ages	*p*-value	Corrected *p*-value		
						0–2 y *vs*. 3–5 y	3–5 y *vs*. 6–13 y	0–2 y *vs*. 6-13 y
*Haemophilus influenzae*								
BLNAS	78 (32.8)	105 (36.1)	55 (32.7)	238 (34.1)				
low–BLNAR	54 (22.7)	64 (22.0)	29 (17.3)	147 (21.1)				
BLNAR	74 (31.1)	86 (29.6)	58 (34.5)	218 (31.3)				
BLPAR	30 (12.6)	33 (11.3)	24 (14.3)	87 (12.5)				
BLPACR	2 (0.8)	3 (1.0)	2 (1.2)	7 (1.0)				
*Streptococcus pneumoniae*								
PSSP	132 (54.8)	162 (69.8)	122 (80.3)	416 (66.6)	<0.0001		0.0002	<0.0001
PISP	100 (41.5)	66 (28.5)	28 (18.4)	194 (31.0)	<0.0001		0.013	0.0007
PRSP	9 (3.7)	4 (1.7)	2 (1.3)	15 (2.4)				

Data are presented as numbers (%) unless indicated otherwise.

BLNAR, β-lactamase-negative ampicillin-resistant; BLNAS, β-lactamase-negative ampicillin-susceptible; BLPAR, β-lactamase-producing ampicillin-resistant; BLPACR, β-lactamase–producing amoxicillin–clavulanic acid–resistant; PISP, penicillin-intermediate *Streptococcus pneumoniae*; PRSP, penicillin-resistant *S. pneumoniae*; PSSP, penicillin-susceptible *S. pneumoniae*.

### 3.4 Age-specific distribution of bacterial isolates

As shown in **Table 4**, the distribution of bacterial species varied markedly by patient age group. *M. catarrhalis* was the most frequently isolated species overall, with particularly high prevalence among younger children. Its detection rate decreased significantly with age: 38.0% in the 0-2-year group, 31.0% in the 3-5-year group, and 21.7% in the 6-13-year group (*p* < 0.0001). *Post hoc* analysis with correction for multiple comparisons revealed significant differences between the 3-5 and 6-13-year groups, as well as between the 0-2 and 6-13-year groups (both corrected *p* < 0.0001). *H. influenzae* was the second most frequently detected species, with detection rates of 22.6%, 25.6%, and 19.9% in the 0-2, 3-5, and 6-13-year groups, respectively. Although a decreasing trend with age was observed, the overall difference among groups was statistically significant (*p* = 0.005). However, no significant differences were found between any of the groups after correction. *S. pneumoniae* showed a similar trend, with detection rates of 22.9% in the 0-2-year group, 20.4% in the 3-5-year group, and 18.0% in the 6-13-year group; however, these differences were not statistically significant. In contrast, *S. aureus* showed the opposite trend, with detection rates increasing with age: 8.3% in the 0-2-year group, 13.4% in the 3-5-year group, and 27.6% in the 6-13-year group. These differences were statistically significant (*p* < 0.0001), and *post hoc* comparisons revealed a significant difference between the 0-2 and 6-13-year groups (corrected *p* < 0.0001). *S. pyogenes* was infrequently detected but showed a slight age-related increase, with rates of 0.6% in the 0–2-year group, 2.6% in the 3–5-year group, and 3.3% in the 6–13-year group. The overall difference among groups was statistically significant (*p* = 0.0003), with a significant difference observed between the 0-2 and 3-5-year groups (corrected *p* = 0.032).

**Table 4:** Bacterial Isolates by age group.

Bacterial species	0–2 y (n = 1054)	3–5 y (n = 1136)	6–13 y (n = 844)	Total (n = 3034)	*p*-value	Corrected *p*-value		
						0–2 y *vs*. 3–5 y	3–5 y *vs*. 6–13 y	0–2 y *vs*. 6-13 y
*Moraxella catarrhalis*	401 (38.0)	352 (31.0)	183 (21.7)	936 (30.9)	<0.0001		<0.0001	<0.0001
*Haemophilus influenzae*	238 (22.6)	291 (25.6)	168 (19.9)	697 (23.0)	0.005			
*Streptococcus pneumoniae*	241 (22.9)	232 (20.4)	152 (18.0)	625 (20.6)				
*Staphylococcus aureus*	88 (8.3)	152 (13.4)	233 (27.6)	473 (15.6)	<0.0001			<0.0001
*Moraxella* spp.	72 (6.8)	74 (6.5)	64 (7.6)	210 (6.9)				
*Streptococcus pyogenes*	6 (0.6)	30 (2.6)	28 (3.3)	64 (2.1)	0.0003	0.032		
*Pseudomonas aeruginosa*	2 (0.2)	2 (0.2)	1 (0.1)	5 (0.1)				
others	6 (0.6)	3 (0.3)	15 (1.8)	24 (0.8)	0.018		0.021	

Data are presented as numbers (%) unless indicated otherwise.

The category “others” included a variety of less commonly isolated organisms such as β-hemolytic *Streptococcus* group G (n = 3), *Acinetobacter* spp. (n = 3), *Streptococcus agalactiae* (n = 3), *Pseudomonas* spp. (n = 2), *Bacillus* spp. (n = 1), *Citrobacter freundii* (n = 1), *Klebsiella pneumoniae* (n = 1), and *Stenotrophomonas maltophilia* (n = 1). Although individually infrequent, these organisms were included in the “others” category due to their potential clinical relevance.

## 4 Discussion

### 4.1 Patient background and specimen characteristics

This study analyzed 2009 nasal discharge specimens collected at otorhinolaryngology outpatient clinics across Japan between 2023 and 2025. Consistent with the epidemiological trends of otorhinolaryngologic infections, the majority of patients were children aged 5 years or younger (0-2 years: 32.6%; 3-5 years: 35.2%; 6-13 years: 32.2%). The bacterial culture positivity rate was high, at 92.7%, indicating that specimens were appropriately collected from patients with suspected bacterial infections.

The most frequently isolated pathogens were *M. catarrhalis*, *H. influenzae*, and *S. pneumoniae*, which together accounted for 74.5% of all isolates. This distribution aligns with previous studies conducted in pediatric populations in Japan. For example, one earlier report found that *H. influenzae* and *S. pneumoniae* were detected in approximately 45% and 32% of pediatric acute maxillary sinusitis cases, respectively (Sawada and Matsubara, 2021), which is generally consistent with the detection rates observed in the present study. Similar bacterial profiles have also been reported in other studies of AOM and rhinosinusitis among Japanese children (Suzuki et al., 2020).

In the present study, the proportion of *M. catarrhalis* isolates was higher than that reported in previous studies. Several factors may account for this finding. First, the elevated detection rate may reflect the characteristics of the study population. This investigation was conducted in otolaryngology outpatient clinics and included children with relatively mild upper respiratory symptoms. In contrast, many prior studies focused on hospitalized patients or those with more severe infections (Hultman Dennison et al., 2019). In such cases, more invasive pathogens such as *S. pneumoniae* and *H. influenzae* tend to predominate, whereas in milder, community-based cases, colonizing organisms like *M. catarrhalis* may be more frequently detected. Second, the use of nasal discharge specimens may have contributed to the higher isolation rate of *M. catarrhalis*. This organism is a well-known colonizer of the upper respiratory tract and is more frequently identified in nasal secretions than in samples such as middle ear fluid or sinus aspirates (Conway et al., 2023). Thus, some of the *M. catarrhalis* isolates identified in this study may represent colonization rather than true pathogenic involvement in sinusitis. Nonetheless, *M. catarrhalis* is also recognized as a potential pathogen in pediatric rhinosinusitis, and clinical interpretation should take into account the patient’s symptoms and the severity of illness when evaluating its significance.

### 4.2 Antimicrobial susceptibility of *H. influenzae* and *S. pneumoniae*

One of the major concerns in this study was the high prevalence of antimicrobial resistance among community-acquired isolates. Among *S. pneumoniae* isolates, PISP accounted for approximately 31.0%, and PRSP for 2.4%. Resistance to clarithromycin was also high at 84.3% overall, with particularly elevated rates among penicillin-non-susceptible strains. These findings are largely consistent with previous nationwide multicenter studies in Japan, which reported that approximately 49.9% of pneumococcal strains from children were penicillin-non-susceptible and over 80% were resistant to macrolides (Kawaguchiya et al., 2024). This resistance trend is likely attributable to the clonal expansion of non-PCV13 serotypes such as 15A, 23A, and 35B, as supported by multiple surveillance studies (Kawaguchiya et al., 2017; Schellenberg et al., 2025). Although serotyping was not performed in this study, based on the timing of sample collection and observed resistance patterns, these non-vaccine serotypes were likely predominant. The introduction of PCV15 in 2023 and PCV20 in 2024 is expected to alter the serotype distribution and antimicrobial resistance patterns of *S. pneumoniae*. However, serotypes 15A and 23A, which are associated with multidrug-resistant community-acquired infections, are not included in PCV20, and infections caused by these serotypes are expected to persist. Similar trends involving emerging serotypes not covered by existing vaccines have also been reported in Western countries (Hoshino et al., 2013; Sheppard et al., 2016), suggesting a shared global challenge. Regarding *H. influenzae*, 44.8% of isolates were resistant to ampicillin, with 31.3% classified as BLNAR, 12.5% as BLPAR, and 1.0% as BLPACR. This distribution is generally consistent with domestic reports from the early 2010s (Barberán et al., 2012). Although the predominance of BLNAR strains has shown a slight improving trend, it still persists, and given the absence of a vaccine targeting non-typeable *H. influenzae*, appropriate antimicrobial stewardship remains the only effective strategy for control.

The MIC data in this study served as a key indicator for characterizing phenotypic resistance. For *S. pneumoniae*, the MIC_50_ and MIC_90_ for penicillin G were 0.06 μg/mL and 1 μg/mL, respectively, with PRSP strains exhibiting MICs exceeding 2 μg/mL. Cefotaxime and cefditoren were highly effective, with MIC_90_ values of 0.5 μg/mL and 0.25 μg/mL, respectively. However, PRSP strains demonstrated reduced susceptibility to cefditoren (MIC_90_ = 1 μg/mL), consistent with previous reports (Brook et al., 1994). For macrolides, the MIC_90_ for clarithromycin exceeded 2 μg/mL, indicating the presence of highly resistant strains, in alignment with nationwide surveillance data (Kawaguchiya et al., 2024). In contrast, levofloxacin and vancomycin exhibited MICs well below clinical breakpoints, supporting their potential utility in refractory cases, as highlighted in earlier studies (Lambert et al., 2025). For *H. influenzae*, the MIC_50_ and MIC_90_ for ampicillin were 2 μg/mL and ≥8 μg/mL, respectively, reflecting the high prevalence of BLNAR and BLPAR strains. Elevated MICs for amoxicillin–clavulanic acid were also observed among BLNAR isolates (MIC_90_ = 8 μg/mL). In contrast, third-generation cephalosporins such as cefotaxime and cefditoren maintained favorable activity, with MIC_90_ values of 1 μg/mL and 0.25 μg/mL, respectively. Clarithromycin MICs reached ≥64 mg/mL in some isolates, indicating limited efficacy of macrolides. Although meropenem showed a noticeable proportion of intermediate isolates among low-BLNAR, BLNAR, and BLPACR strains, there was no marked increase in MIC values (MIC_90_ 0.25 mg/mL), supporting its role as a reliable therapeutic option in complicated or resistant cases. These MIC distributions corroborate observed phenotypic resistance patterns and underscore the clinical importance of selecting antimicrobials based on *in vitro* susceptibility, particularly in regions such as Japan, where resistant pathogens are highly prevalent.

Notably, compared with earlier reports from the 2010s, the proportion of BLNAR strains, while still high, appears to have declined slightly. For example, a study conducted between 2013 and 2016 reported that BLNAR strains accounted for over 50% of pediatric isolates, whereas the present study suggests a modest reduction. This shift may reflect the sustained impact of improved antimicrobial stewardship and reduced selective pressure following the 2013 revision of the pediatric AOM guidelines. Moreover, the 2024 guideline revision recommends high-dose amoxicillin and careful observation for mild cases, which may further influence future resistance trends (The Japanese Society for Pediatric Otorhinolaryngology, 2024). Indeed, countries such as the Netherlands and Sweden have reported decreased resistance rates following similar changes in antibiotic use policies (Takeuchi et al., 2017; Eriksen et al., 2021), and comparable benefits may also be anticipated in Japan.

In this study, the overall susceptibility of *S. pneumoniae* and *H. influenzae* to levofloxacin was not exceptionally high, with MIC_90_ values of 1 μg/mL and ≤0.25 μg/mL, respectively. However, a small number of isolates exhibited elevated MICs, raising concerns about the potential emergence of resistance with increasing pediatric use of fluoroquinolones. Since 2010, tosufloxacin has been approved as an oral fluoroquinolone for children in Japan and has been used in limited cases (Takeuchi et al., 2021). Although quinolone resistance among respiratory pathogens remains uncommon, *H. influenzae* strains harboring mutations in the *gyrA* and *parC* genes have been reported, indicating early warning signs of emerging resistance (Kovács et al., 2021). Furthermore, between 2023 and 2024, Japan experienced supply shortages of key β-lactam antibiotics such as amoxicillin and cefdinir, temporarily limiting access to first-line agents. As a result, increased use of fluoroquinolones was reported in some clinical settings, potentially heightening selective pressure for resistance. While fluoroquinolones remain clinically effective at present, their therapeutic margin is narrow, underscoring the need for continued surveillance. From an antimicrobial stewardship perspective, the use of fluoroquinolones in children should be restricted to specific indications—such as β-lactam allergy or infections caused by treatment-refractory pathogens—in accordance with current pediatric infectious disease guidelines (The Japanese Society for Pediatric Otorhinolaryngology, 2024).

It is also necessary to discuss the appropriateness of macrolide monotherapy in pediatric sinusitis. Although macrolides have historically been used as empirical therapy, their clinical efficacy against the major causative pathogens of sinusitis is limited. *S. pneumoniae*, one of the principal pathogens, frequently exhibits macrolide resistance, and recent surveillance data from Japan have reported resistance rates exceeding 80% (Nagai et al., 2014). Moreover, macrolides generally exert bacteriostatic rather than bactericidal activity, which may be insufficient for pathogen eradication in infections with a high bacterial burden. For these reasons, both international and domestic guidelines recommend β-lactam antibiotics as the first-line therapy, reserving macrolides only for patients with severe β-lactam allergy. Therefore, macrolide monotherapy should be avoided as an empirical treatment for pediatric sinusitis.

### 4.3 Age-specific distribution of bacteria

Age-stratified analysis revealed clear differences in the distribution of bacterial species. *M. catarrhalis*, *H. influenzae*, and *S. pneumoniae* were predominantly detected in children aged ≤5 years, reaffirming their roles as major pathogens in pediatric rhinosinusitis. In contrast, the detection frequency of *S. aureus* increased with age, reaching 27.6% in the 6–13-year age group. This trend may be associated with factors such as a higher prevalence of chronic rhinosinusitis during adolescence and age-related changes in the nasal microbiota (Kishita et al., 2023). Additionally, 22% of the *S. aureus* isolates were identified as MRSA, most of which are presumed to be community-acquired strains. MRSA remains a clinically significant pathogen in otorhinolaryngology, and national surveillance data have consistently reported its involvement in community-acquired infections (Suzuki et al., 2020; Silva-Costa et al., 2024). Furthermore, MRSA involvement in refractory rhinosinusitis among both children and adults is reportedly increasing (Lambert et al., 2025). In such difficult-to-treat cases, culture-based diagnosis and susceptibility-guided antimicrobial therapy are essential for effective management.

The study period coincided with the post– coronavirus disease 2019 pandemic era, during which mask-wearing and the suppression of respiratory viral infections may have temporarily influenced the dynamics of bacterial infections. Indeed, an increase in outpatient visits and nasal discharge cultures was observed after the pandemic, possibly reflecting a return to typical seasonal patterns of respiratory infections. A similar “post-pandemic rebound” in bacterial infections following the resurgence of viral illnesses has also been reported in European pediatric respiratory surveillance studies (Silva et al., 2021). Furthermore, the increased detection rate of MRSA in adolescents may be related to cumulative antibiotic exposure and alterations in nasal flora associated with changes in hygiene practices. These age-related differences in pathogen distribution provide important insights for empirical antimicrobial selection. In particular, in older children or in cases of recurrent or treatment-resistant rhinosinusitis, MRSA should be considered, and individualized treatment based on culture and susceptibility testing is recommended if β-lactam therapy fails. To support and refine such treatment strategies, continued region-specific microbiological surveillance is critically important.

## 5 Limitations and future directions

This study demonstrated that *H. influenzae* and *S. pneumoniae* remain the predominant pathogens in pediatric chronic rhinosinusitis, while the clinical significance of *S. aureus*, particularly MRSA, is increasing in older children. Given the high resistance rates of *S. pneumoniae* to penicillin and macrolides, empirical treatment with macrolide monotherapy is not recommended. In contrast, amoxicillin–clavulanic acid remains a reasonable treatment option due to its effectiveness against *H. influenzae*, *M. catarrhalis*, and many strains of *S. pneumoniae*. Furthermore, in older children and in cases of recurrent or refractory rhinosinusitis, the potential involvement of *S. aureus*—especially MRSA—should be taken into consideration.

The limitations of this study include its single-center design, the absence of serotyping or genotyping, and the lack of clinical differentiation between acute and chronic cases. In addition, the absence of data on prior antibiotic exposure—which may represent a potential confounding factor for resistance rates—the lower specificity of nasal discharge cultures compared with sinus aspirate cultures in reflecting true infection, and the lack of information on adherence to treatment guidelines should also be acknowledged as limitations of this study. Nevertheless, the findings are consistent with existing national surveillance data and are considered to have a reasonable degree of generalizability. Looking ahead, multicenter molecular epidemiological studies are needed to evaluate the impact of the introduction of PCV15 and PCV20, as well as adherence to pediatric clinical guidelines, on trends in antimicrobial resistance. In addition, a comprehensive assessment of host factors, underlying medical conditions, and vaccination history will be essential for further advancing clinical understanding and management.

## 6 Conclusion

This study provides timely and clinically relevant data on the microbiological profile of pediatric acute and chronic rhinosinusitis during a transitional period marked by the introduction of new vaccines and revisions to antibiotic prescribing guidelines. Notably, the predominance of *H. influenzae* and *S. pneumoniae* in young children, along with the increasing clinical relevance of *S. aureus* in older children, offers practical insights for age-specific empirical therapy. Furthermore, the high prevalence of penicillin- and macrolide-resistant *S. pneumoniae*, as well as persistent ampicillin resistance in *H. influenzae*, highlights the importance of appropriate antibiotic use. Clinically, these findings support rational antibiotic selection in pediatric otorhinolaryngologic care, advocate for culture-based management in recurrent or refractory cases, and emphasize the need to consider MRSA in adolescents and cases of chronic rhinosinusitis. Ongoing microbiological surveillance remains essential for tracking resistance trends and guiding regionally appropriate treatment strategies.

## Data Availability

The original contributions presented in the study are included in the article/supplementary material. Further inquiries can be directed to the corresponding author.
